# Efficacy of photodynamic therapy in the treatment of oral candidiasis: a systematic review and meta-analysis

**DOI:** 10.1186/s12903-023-03484-z

**Published:** 2023-10-26

**Authors:** Qiaoyu Hu, Ting Li, Jiadi Yang, Yanhui Peng, Qing Liu, Na Liu

**Affiliations:** 1 Hebei Key Laboratory of Stomatology, Hebei Clinical Research Center for Oral Diseases, School and Hospital of Stomatology, Hebei Medical University, Shijiazhuang, 050017 China; 2 Department of Preventive Dentistry, School and Hospital of Stomatology, Hebei Medical University, No. 383, Zhongshan East Road, Shijiazhuang, 050017 PR China

**Keywords:** Oral candidiasis, Candida, Photodynamic therapy, Photochemotherapy, Meta-analysis

## Abstract

**Objective:**

To evaluate the clinical efficacy of photodynamic therapy (PDT) as an adjunct or alternative to traditional antifungal drugs in the treatment of oral candidiasis, and to provide evidence-based medical evidence for its use in the treatment of oral candidiasis.

**Methods:**

Computer combined with manual retrieval of China Academic Journals Full-text Database (CNKI), China Biomedical Literature Database (CBM), Chinese Science and Technology Journal Database (VIP), Wanfang Database, PubMed, Web of Science, Cochrane Library, Embase, Scopus retrieval for articles published before January 2023, basic information and required data were extracted according to the inclusion and exclusion criteria, and the Revman V5.4 software was used to conduct Meta-analysis of the included literature.

**Results:**

A total of 11 articles were included, 7 of which used nystatin as an antifungal drug, 2 of which were combined treatment of PDT and nystatin, 2 of the remaining 4 articles were treated with fluconazole, and 2 were treated with miconazole. Meta results showed that PDT was superior to nystatin in reducing the number of oral candida colonies in the palate of patients *MD* = *-0.87, 95%CI* = *(-1.52,-0.23), P* = *0.008*, the difference was statistically significant, and the denture site *MD* = *-1.03, 95%CI* = *(-2.21, -0.15), P* = *0.09*, the difference was not statistically significant; compared with the efficacy of fluconazole, *RR* = *1.01, 95%CI* = *(0.56,1.83), P* = *0.96*; compared with miconazole *RR* = *0.55, 95%CI* = *(0.38, 0.81), P* = *0.002*; PDT combined with nystatin *RR* = *1.27, 95%CI* = *(1.06, 1.52), P* = *0.01*; recurrence rate *RR* = *0.28, 95%CI* = *(0.09, 0.88), P* = *0.03*.

**Conclusions:**

PDT was effective in the treatment of oral candidiasis; PDT was more effective than nystatin for the treatment of denture stomatitis in the palate, while there was no significant difference between the two for the denture site; The efficacy of PDT for oral candidiasis was similar to that of fluconazole; PDT was less effective than miconazole for oral candidiasis; Compared with nystatin alone, the combination of PDT and nystatin is more effective in treating oral candidiasis with less risk of recurrence.

**Supplementary Information:**

The online version contains supplementary material available at 10.1186/s12903-023-03484-z.

## Introduction

Oral candidiasis (OC) is a fungal infectious disease of the oral mucosa caused by Candida [1]. In recent years, with the application of broad-spectrum antibiotics, glucocorticoids, and immunosuppressants, organ transplantation, and tumor treatment, the number of patients with impaired immune function has increased, and the incidence of oral candidiasis has continued to increase, becoming the most common infectious disease of the oral mucosa [2]. Patients with oral candidiasis are often associated with burning, unpleasant bitter or salty taste, altered taste, and sometimes pain and discomfort, difficulty swallowing, nausea, vomiting, and diarrhea. These symptoms may affect the patient's ability to eat and lead to changes in the patient's quality of life [3].

The current effective treatments for oral Candida infections are topical and systemic treatments. For mild cases of infection, topical antifungal drugs such as nystatin are recommended [4]. The treatment period of nystatin is long, usually 14–28 days or even longer, with occasional adverse effects such as nausea, diarrhea or loss of appetite after taking it [5]. For patients who are immunocompromised or at risk of disseminated candidiasis, systemic antifungal therapy, such as azoles, can be used [6]. However, the increasing use of azoles has led to an increase in Candida resistance to antifungal drugs [7], necessitating the search for new therapeutic approaches. Alternative antifungal drugs that have been considered include colloidal solutions of metal nanoparticles (silver, gold), ozone therapy, photo biomodulation and photodynamic therapy, of which photodynamic therapy is a promising new therapy [8].

Photodynamic therapy (PDT) is a treatment method in which a photosensitizer is applied to the diseased tissue and a photochemical reaction is produced by irradiation with a specific wavelength light source to achieve a therapeutic effect. Reactive oxygen species (ROS) produced in photochemical reactions can react with a variety of biomolecules such as phospholipids, nucleic acids and proteins of cells to produce toxicity thereby inactivating cells and other microorganisms [9–11]. In recent years, as PDT continues to be used in clinical practice, its anti-Candida effect has received increasing attention. The main benefits of PDT over conventional antifungal therapy include its broad antimicrobial spectrum, short therapeutic course, strong targeting, high selectivity, and low impact on surrounding normal tissue cells [1]. Additionally, studies have shown that Candida is susceptible to photoinactivation, including drug-resistant strains, which can treat recurrent infections brought on by drug-resistant Candida [12–14]. Therefore, PDT shows potential applications in the treatment of oral infections.

The light source chosen for PDT is usually a low-energy laser with a power of less than 500mW and a wavelength range of 630 ~ 750 nm, which can be precisely regulated in terms of output power and produces only a localized effect, and therefore does not require any special protection and will not burn the surrounding tissues [15]. A variety of light sources have been used for PDT therapy, and semiconductor lasers, which have the advantages of being easy to operate, portable, and cost-effective, have been more and more widely used; in addition, non-laser light sources, such as incandescent lamps, quartz halogen lamps, and light-emitting diodes, have been used to a certain extent as well [16]. Photosensitizer is an important factor in the successful application of photodynamic therapy, commonly used in the clinic is the second generation of photosensitizers, mostly porphyrin compounds derivatives, such as 5-amino ketoglutaric acid; as well as stains and dyes, such as bracketed toluidine blue, methylene blue, rose red, erythrosine, and peacock green, which have strong photoinactivation effect [17]. Photosensitizers increase the inhibition rate with incubation time, reaching a peak plateau at 30 ~ 90 min [18].

The current clinical effectiveness of PDT for oral candidiasis is variable, and Mima [19] and Senna [20] conducted a randomized controlled trial of PDT for oral candidiasis comparing the efficacy of PDT with that of mycophenolate and found no significant difference between the two in terms of clearance of oral Candida, but the PDT group required a shorter course of treatment to achieve the same effect. Maciel [21] et al. compared PDT combined with a low-energy laser with miconazole gel in the treatment of oral Candida and showed that although the PDT group had some efficacy, its cure rate was significantly lower than that of the miconazole group. Therefore, this study used Meta-analysis to systematically evaluate PDT for the treatment of oral Candida and to provide a basis for clinical application.

Different from previous systematic reviews, this study is not limited to single-drug control and outcome indicators, but compares PDT with nystatin, fluconazole, and miconazole, and more comprehensively evaluates the effects of PDT and antifungal drugs on oral candida. disease treatment effect. The possible mechanisms of PDT in combination with nystatin for the treatment of oral candidiasis were also explored, as well as the recurrence after treatment and the safety of the treatment approach.

## Materials and methods

This Meta-analysis is based on the PRISMA 2020 Statement: Updated Guidelines for the Reporting of Systematic Reviews guidance [22] for asking questions, registered with INPLASY (registration number INPLASY2022120053), asking “Is photodynamic treatment of oral Candida effective compared to conventional antifungal drugs?”.

### Inclusion criteria

The criteria for inclusion in the study were based on the PICOS strategy.

P: Patients diagnosed with oral candidiasis (e.g., denture stomatitis, HIV with Candida infection)

I: PDT of any type of light source and photosensitizer

C: Use of topical or systemic traditional antifungal drugs (e.g., nystatin, fluconazole)

O: Primary outcomes included a reduction in the number of Candida colonies in the patient’s palate and denture or resolution of inflammation in the palate.

S: Randomized Controlled Trial.

Exclusion criteria:

- 1. Documents in languages other than English and Chinese
- 2. Duplicate literature
- 3. Studies unable to provide original data

### Search strategy

A combination of computer and manual searches were conducted electronically for literature published in the Chinese Academic Journal Full Text Database (CNKI), Chinese Biomedical Literature Database (CBM), Chinese Science and Technology Journal Database (VIP), Wanfang Database, PubMed, Web of science, Cochrane Library, and Embase as of January 2023. A manual search was also conducted for the incorporated literature of relevant systematic evaluations. Combination of subject terms and free words according to Boolean logic operation, Search terms are as follows:

- Photochemotherapy OR Photochemotherapies OR Photodynamic Therapy OR Therapy, Photodynamic OR Photodynamic Therapies OR Therapies, Photodynamic OR Antibacterial photodynamic therapy OR Photodynamic antimicrobial chemotherapy OR Photodynamic inactivation OR PDT OR APDT OR PAD OR PDI
- Candida OR Candidiasis OR Candidiases OR Candidiasis, Oral OR Candidiases, Oral OR Oral Candidiases OR Oral Candidiasis OR Thrush OR Moniliasis, Oral OR Moniliases, Oral OR Oral Moniliases OR Oral Moniliasis OR Stomatitis, Denture OR Denture Stomatitides OR Denture Stomatitis OR Stomatitides, Denture OR AIDS-related oral candidiasis randomized controlled trial OR randomized OR placebo

### Literature screening

Screening and study selection were independently performed by two researchers, all records were imported into the literature management software, duplicate literature was deleted, and the titles and abstracts of all retrieved literature were pre-screened for potentially eligible studies. A detailed assessment was then carried out according to pre-determined eligibility criteria for inclusion in the review. Disagreeing documents were resolved through consultation or with the assistance of relevant experts, and the final decision was made on whether to include them until a consensus was reached.

### Data extraction

The following data were obtained from the included studies: author, publication year, study design, sample size, light source characteristics, pre-irradiation time, photosensitizer type, antifungal drug characteristics, follow-up period and safety, etc. Two researchers performed the data collection process independently. Collect data electronically using Excel sheets.

### Literature quality evaluation

The risk of bias of each study was assessed according to the Cochrane Handbook for Systematic Reviews of Interventions, version 5.1.0 [23], including: method of randomization, concealment of allocation, blinding of participants and researchers, blinding of outcome assessments, completeness of outcome data, selective publication, and other sources of bias. The above results were assessed by two researchers for the quality of the included literature, and inconsistencies were discussed and determined by the superior physician.

### Efficacy evaluation index

The indicators for evaluating the efficacy of oral candidiasis include clinical evaluation and microbiological evaluation [24], clinical evaluation refers to the clinical improvement of patients after treatment, the microbiological evaluation focuses on the effectiveness of this treatment measure by assessing the change in Candida colony forming units per milliliter (CFU/ml).

### Statistical analysis

Meta-analysis of the included literature was performed using Review Manager (Revman, version 5.4, Cochrane Community) software, In this study, the clinical efficacy is an ordered classification data(Ineffective, Effective, Significant, Cured), which is converted into a binary classification variable for analysis (i.e., ineffective vs. total effective, total number of effective = effective + significantly effective + cured), and the RR value and 95% confidence interval are used to indicate the size of the effect; Colony forming unit (CFU) is a continuous variable, which is grouped and analyzed according to follow-up time, and the effect size is represented by mean difference MD and 95% confidence interval (CI); A random-effects model was used for the analyses, taking into account the large clinical variation between population and treatments among studies.

### GRADE quality of evidence assessment

Referring to the GRADE quality of evidence grading system [25] to grade the evidence for outcome indicators, which contains 5 downgrading factors and 3 upgrading factors, the literature included in this study were all RCTs with the highest level of evidence, so they were not upgraded. The quality of evidence was categorized into 4 levels of High, Moderate, Low, and Very Low based on the 5 dimensions of risk of bias, inconsistency, indirectness, imprecision, and publication bias.

## Results

### Literature search and screening results

**Fig. 1:**
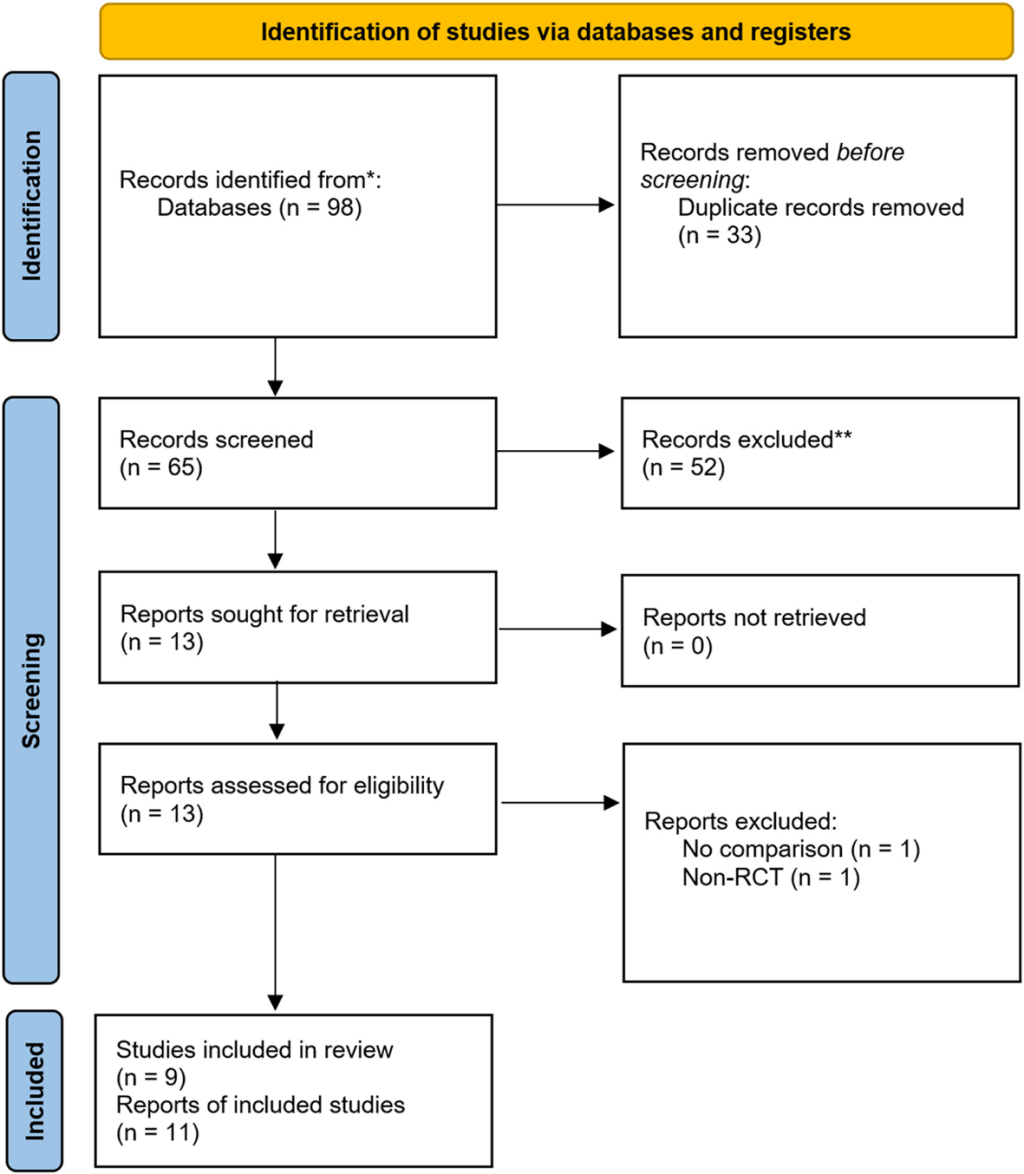
Flow chart of search

Through a combined computer and manual search, 98 literatures were initially retrieved, 33 duplicates were removed, a total of 52 were excluded after further reading of the title and abstract, and 2 were excluded after reading the full text, and finally a total of 11 literatures met the inclusion criteria, as shown in Fig. 1.

### Characteristics of the included literature

In total, there were 11 literatures, 7 of which the antifungal drug was nystatin, 2 of which were PDT in combination with nystatin, and 2 of the remaining 4 were fluconazole treatment and 2 were miconazole treatment (Table 1).

**Table 1:** Basic characteristics of the included literature

Author/Year	Design	Type	Average Age		Gender (F/M)		Denture Age		Smoking Number of people		Sample size		Group	Treatment Area	Antifungal treatment	
			Antifungal	PDT	Antifungal	PDT	Antifungal	PDT	Antifungal	PDT	Antifungal	PDT			Drug Type	Treatment Time/Total Days/Usage per Day
Labban 2021 [26]	RCT	DS	56.9	57.2 59.5	11/4	12/3 10/5	13.5	14.5 16.7	15	15 15	15	15 15	G1- PDT(Rb) G2- PDT(Cur) G3- antifungal	Palate Denture	Nystatin 100,000IU	1min/15/4
Alves 2020 [27]	RCT	DS	69	70	24/9	19/11	6	7	3	5	33	30	G1-PDT G2-antifungal	Palate Denture	Nystatin 100,000IU	1min/15/4
Alrabiah 2019 [28]	RCT	DS	-	-	-	-	-	-	-	-	18	18	G1- PDT G2- antifungal	Palate Denture	Nystatin 100,000IU	1min/15/4
Mima 2012 [19]	RCT	DS	62.45	61.25	15/5	13/7	18.55	14	2	4	20	20	G1- PDT G2- antifungal	Palate Denture	Nystatin 100,000IU	1min/15/4
C Chen 2022 [29]	RCT	OC	52.18	52.25	25/18	24/19	-	-	-	-	43	43	G1- PDT + antifungal G2- antifungal	Palate	Nystatin 500,000IU	-/7/3
Afroozi 2019 [30]	RCT	DS	67.4	67.6	21/7	22/6	-	-	-	-	33	33	G1- PDT + antifungal G2- antifungal	Palate	Nystatin 100,000IU	4min/15/3
Senna 2018 [20]	RCT	DS	54.7	58.1	17/1	17/1	-	-	1	3	18	18	G1- PDT G2- antifungal	Palate Denture	Miconazole gel 2%	-/30/3
Y Zhao 2018 [31]	RCT	HIV infection with OC	47.06	54.57	7/11	12/30	-	-	-	-	18	42	G1- PDT G2- antifungal	Oral mucosa	Nystatin + 5% NaHCO_3_	10-20ml, 5-6min, 2 times a day
JM Qin 2018 [32]	RCT	HIV infection with OC	-	-	-	-	-	-	-	-	9	9 9	G1- PDT + KI (200μmol MB) G2- PDT + KI (400μmol MB) G3- antifungal	Oral mucosa	Fluconazole 100mg	-/7/1
Maciel 2016 [21]	RCT	DS	51.7	51.2	16/4	14/6	7.1	9.5	-	-	20	20	G1- PDT + LPL G2- antifungal	Palate	Miconazole gel 2%	-/15/4
Scwingel 2012 [33]	RCT	HIV infection with OC	30		5/16		-	-	-	-	7	7	G1- PDT G2-LLLT G3- antifungal	Oral mucosa	Fluconazole 100mg	-/14/1

Author/Year	PDT treatment										
	Light Source/Number/ Wavelength (nm)	Newton’s criteria (Type I, II, III)	Photosensitizer Agent	Pre-irradiation Time	Light intensity(mW/cm^2^)	Energy Density(J/cm^2^)	Clinical evaluation	Microbiological evaluation	Follow-up time	Recurrence	Security
Labban 2021	Palate LED/10/455nm Denture LED/24/455nm	Newton’s criteria (Type I, II, III)	Rb Cur	30min	Palate 102 Denture 24	Palate 122 Denture 37.5	Newton’s criteria (Type I, II, III)	Fungal count	6/12 weeks	-	-
Alves 2020	Palate LED/10/660nm Denture LED/24/660nm	Clinical efficacy	PDZ	20min	Palate 240 Denture 50	Palate 50 Denture 50	Newton’s criteria (Type I, II, III)	Fungal count	15/30/45/60 days	-	-
Alrabiah 2019	GaAlAs laser /-/660nm	Newton’s criteria (Type I, II, III)	MB	10min	-	Palate 28 Denture NA	Clinical efficacy	Fungal count	15/30/60 days	-	-
Mima 2012	Palate LED/10/455nm Denture LED/24/455nm	Clinical efficacy	Hematoporphyrin derivatives	30min	Palate 102 Denture 24	Palate 122 Denture 37.5	Newton’s criteria (Type I, II, III)	Fungal count	15/30/60/90 days	During follow-up, 75% and 78% of patients in the NYT and PDT groups, respectively, had recurrence of palatal inflammation	-
C Chen 2022	LED/-/635nm	Newton’s criteria (Type I, II, III)	DMMB	30min	-	10	Clinical efficacy	Fungal count	Post-treatment sampling /6 months	At 6 months follow-up, 10 recurrences (23.26%) in the control group and 3 recurrences (6.98%) in the study group	1 case of abdominal pain and 1 case of nausea in the control group; 2 cases of nausea, 1 case of diarrhea and 1 case of abdominal pain in the study group
Afroozi 2019	Diode laser/-/ 810nm	Budtz-Jorgensen Classification	ICG	10min	-	56	Newton’s criteria (Type I, II, III)	Fungal count	15/60 days	Only 2 cases of recurrence in the control group	-
Senna 2018	GaAlAs laser/-/ 660nm	Clinical efficacy	MB	10min	-	Palate28 Denture28	Budtz-Jorgensen Classification	Olsen Method	30 days	-	-
Y Zhao 2018	LED/-/635nm	Clinical efficacy	DMMB	5min	-	10	Clinical efficacy	Fungal count	Post-treatment sampling	-	5 cases of nausea and vomiting in the control group and 7 cases of pungency on the back of the tongue in the study group
JM Qin 2018	LED/-/635nm	Newton’s criteria (Type I, II, III)	MB	G1 20min G2 5min	-	10	Clinical efficacy	Fungal count	Post-treatment sampling	-	A total of 3 cases of nausea and 4 cases of burning sensation in the tongue in both study groups
Maciel 2016	GaAlAs laser/-/ 660nm	Clinical efficacy	MB	5min	100	PDT: 1 LPL: 70	Newton’s criteria (Type I, II, III)	-	15 days	At 15 days after the end of treatment, 25% of patients in the experimental group and 12.5% of patients in the miconazole group relapsed	No adverse reactions reported
Scwingel 2012	Twin laser/-/ 660nm		MB	1min	-	7.5	Clinical efficacy	The results were scored as low, moderate and abundant growth based on turbidity classified as clear, mild and intense after incubation	7/15/30 days	On day 30 of the control group, 72% of patients showed recurrence of signs and symptoms, while all patients in the PDT group showed improvement in clinical symptoms	-

*PDT* Photodynamic Therapy, *OC* Oral Candidiasis, *DS* Denture stomatitis, *LPL* low-power laser, *LLLT* Low-level laser therapy, *KI*: Potassium Iodide, *RB* Rose Bengal, *Cr* Curcumin, *PDZ* Photosensitive Diazine, *MB* Methylene Blue, *DMMB* Dimethyl Methylene Blue, *ICG* Indocyanine Green

### Literature quality evaluation

**Fig. 2:**
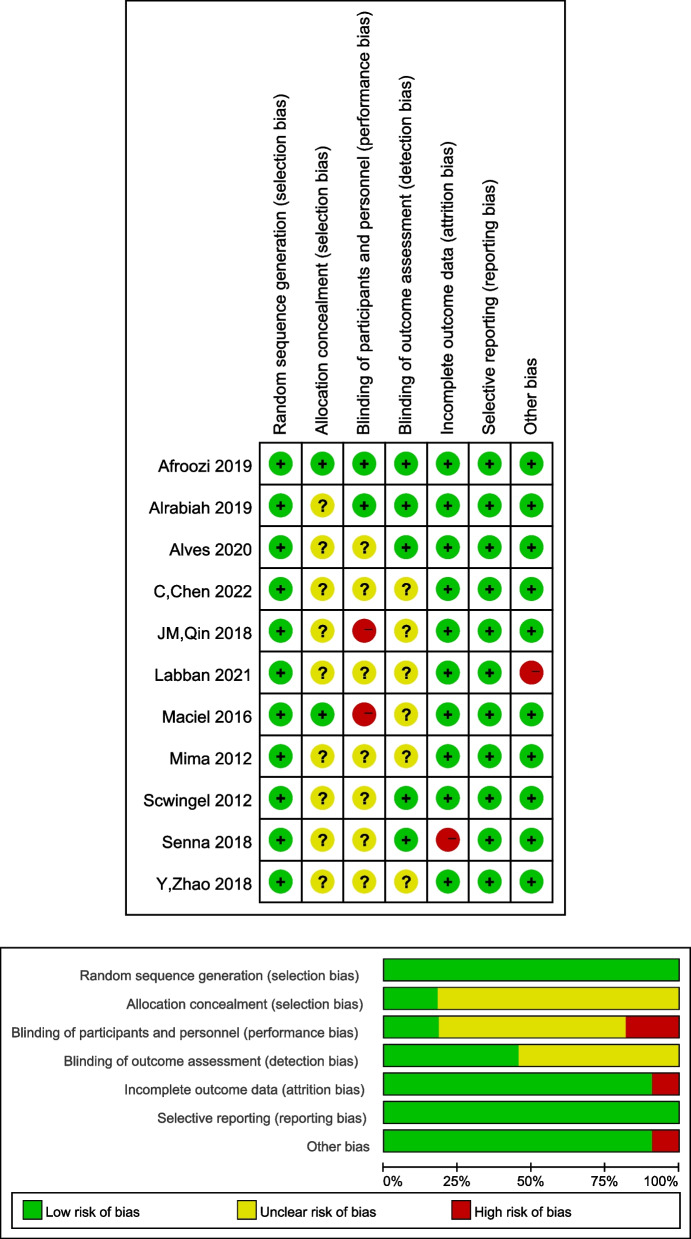
Risk bias graph of the included literature

Eleven studies used randomized controlled methods, of which two implemented allocation protocol concealment, two clearly described blinding of patients and principal investigators, two did not, and five were blinded to the study outcome measure. The results of the quality evaluation of the studies are shown in Fig. 2.

### Meta-analysis results

#### Comparison of PDT and nystatin

**Fig. 3:**
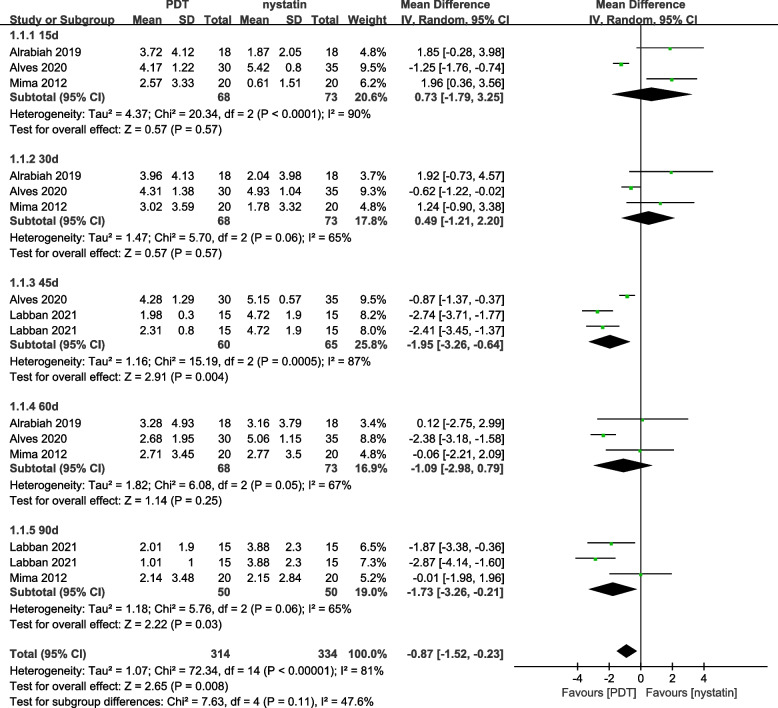
Palatal forest plot of PDT vs. nystatin. (Random effects model)

Palatal efficacy assessment: a total of 4 studies were included, and heterogeneity test showed heterogeneity among studies *(P* < *0.00001, I*^*2*^ = *81%)*, and the random effect model was used for Meta analysis: *MD* = *-0.87, 95% CI* = *(-1.52, -0.23)*, MD combined with 95% CI horizontal line to the left of the null vertical line. Therefore, it can be concluded that PDT cleared more colonies of oral Candida compared to nystatin, with a statistically significant different (*Z* = *2.65, P* = *0.008*), Fig. 3.

**Fig. 4:**
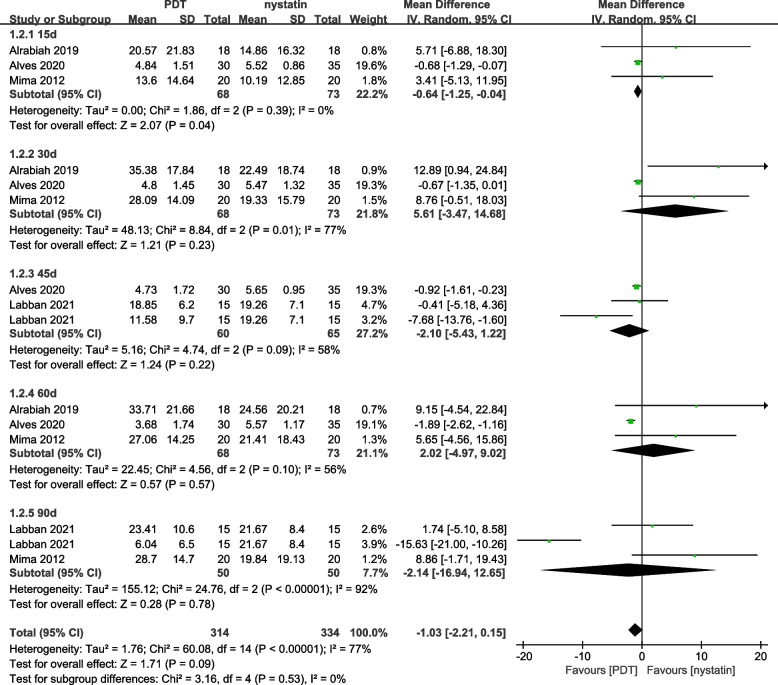
Denture sites forest plot of PDT vs. nystatin (Random effects model)

Denture site efficacy assessment: a total of 4 studies were included and heterogeneity test showed heterogeneity among studies (*P* < *0.00001, I*^*2*^ = *77%*), and the random effect model was used for Meta analysis: *MD* = *-1.03, 95% CI* = *(-2.21, -0.15)*, the 95% CI horizontal line for MD combined was on the null line, the difference was not statistically significant (*Z* = *1.71, P* = *0.09*), Fig. 4.

#### Comparison of PDT and fluconazole

**Fig. 5:**
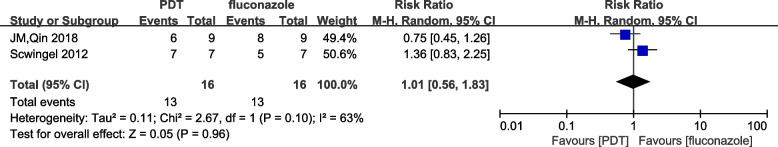
Forest plot of PDT vs. fluconazole (Random effects model)

Efficacy assessment: 2 studies were included, heterogeneity test showed heterogeneity between studies *(P* = *0.10, I*^*2*^ = *63%)*, and the random effect model was used for Meta analysis: *RR* = *1.01, 95% CI* = *(0.56,1.83)*, RR combined with 95% CI horizontal line on the null line *(Z* = *0.05, P* = *0.96)*, the difference was not statistically significant. PDT has similar efficacy to fluconazole in the treatment of oral candidiasis, Fig. 5.

#### Comparison of PDT and miconazole

**Fig. 6:**
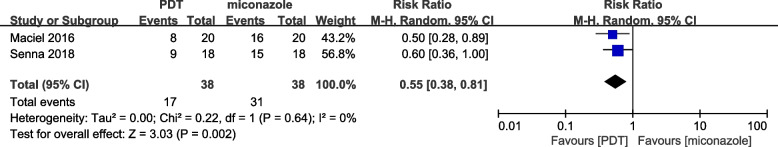
Forest plot of PDT vs. miconazole (Random effects model)

Efficacy assessment: a total of 2 studies were included, the random effect model was used for Meta analysis: *RR* = *0.55, 95% CI* = *(0.38,0.81)*, RR combined with 95% CI horizontal line on the left side of the null line, the results showed that PDT was less effective in treating oral candidiasis than miconazole *(Z* = *3.03, P* = *0.002)*, Fig. 6.

#### PDT + nystatin combination therapy

**Fig. 7:**
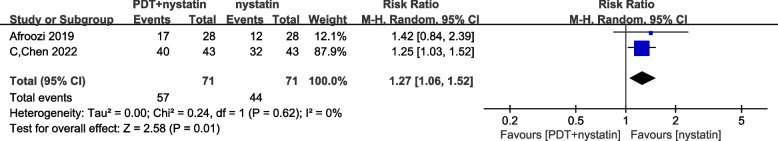
Forest plot of efficacy of PDT + nystatin combination therapy (Random effects model)

Efficacy assessment: 2 studies were included, the random effect model was used for Meta analysis: *RR* = *1.27, 95% CI* = *(1.06, 1.52)*, 95% CI horizontal line for RR combined was to the right of the vertical line of ineffectiveness, the difference was statistically significant (*Z* = *2.58, P* = *0.01*), sufficient evidence exists to suggest that the combination of PDT + mycobacterium is more effective in the treatment of oral candidiasis compared to mycobacterium alone, Fig. 7.

**Fig. 8:**
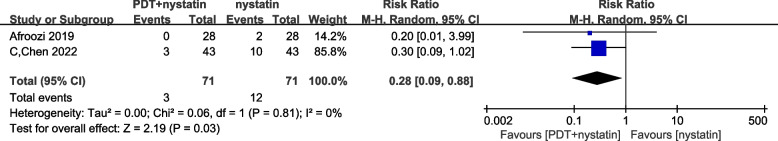
Forest plot of recurrence rate for PDT + nystatin combination treatment (Random effects model)

Recurrence rate: 2 studies were included, the random effect model was used for Meta analysis: *RR* = *0.28, 95% CI* = *(0.09, 0.88)*, RR combined with 95% CI horizontal line located to the left of the null vertical line, results showed that compared to mycobacterium toxin alone, PDT + nystatin combination for oral candidiasis had a lower recurrence rate with a statistically significant difference (*Z* = *2.19,P* = *0.03*), Fig. 8.

#### Evaluation of the quality of GRADE evidence

**Fig. 9:**
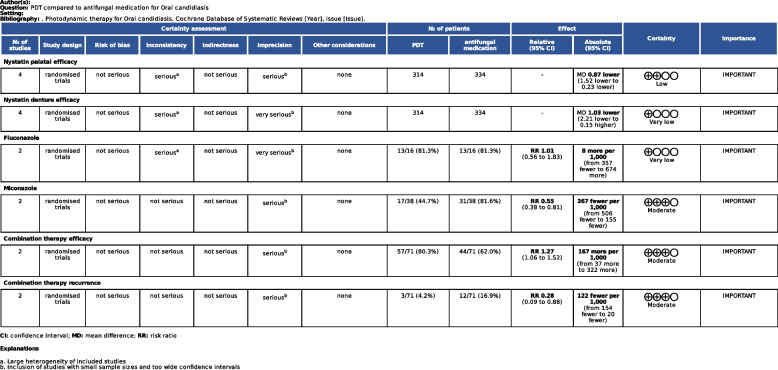
GRADE evidence quality assessment

The quality of evidence was classified using the GRADEpro GDP software, which showed that the efficacy of nystatin palate was of low quality of evidence (Low), the efficacy of nystatin denture site was of very low quality of evidence (Very low), the efficacy of fluconazole was of low quality of evidence (Low), the efficacy of miconazole was of moderate quality of evidence (Moderate), and the efficacy of the combination of PDT + nystatin as well as recurrence rate was of moderate quality of evidence (Moderate), Fig. 9.

## Discussion

The results of this meta-analysis showed that compared with nystatin, when the treatment area was on the palate, there was a statistically significant difference in the reduction of Candida colonies between the two groups (*P* < *0.05*), suggesting that PDT is more effective in removing oral Candida in the palate, but there was no statistically significant difference in the denture area. The process of microbial adhesion to the denture surface is related to the surface properties of the material, such as hydrophobicity and surface roughness [34], and the porous structure and irregular inner surface of the acrylic resin denture act as a reservoir for microorganisms, as well as difficulties in hygienic maintenance and disinfection [35], resulting in more rapid regeneration of Candida in the patient's denture than in the treated palate [36]. The use of PDT technique in the study affected only the palatal mucosa, while treatment with drugs could act on other areas such as the oral mucosa and the tongue, possibly due to dilution of saliva resulting in a weakened effect on Candida and the penetration of the mycelium into the epithelial cells before starting colonization of the palate within 48 h [37]. All of these reasons may cause PDT to be more effective on the palate than on the denture site. The role of PDT in reducing Candida counts is insufficient if the denture surface is not mechanically cleaned [38]. Other topical medications such as nystatin can be used for treatment, but the movement of the oral muscles makes it difficult to keep the medication in the treated area thus reducing the level of treatment [27]. However, systemic antifungals like amphotericin B can be used, but they are not very effective in removing fungal colonies from the surface of the denture [39]. Therefore, it is recommended that all wearers clean all surfaces of the denture regularly to minimize denture-related fungal infections [38]. At the 15-day follow-up of the denture site, there was a statistically significant difference in the reduction of Candida colonies between the two groups, but there was no statistically significant difference at 30 and 60 days, which may be due to the recolonization of Candida on the denture surface after treatment.

Azoles are commonly used in the treatment of Candida infections, but with the massive and unregulated use of antifungal drugs, the resistance rate of Candida to azoles is now gradually increasing, and the phenomenon of cross-resistance between azoles is obvious [40]. Mechanisms of Candida resistance to azoles include altered drug targets, overexpression of drug efflux pumps, altered metabolic pathways and initiation of adaptive stress responses. Mutation or overexpression of the azole target enzyme gene ERG11 in Candida albicans maintains target enzyme activity and produces drug resistance [41]. The results of this Meta showed that the efficacy of PDT in oral candidiasis was similar to that of fluconazole and that miconazole was superior to PDT, but considering that the increasing use of azoles has led to an increase in the resistance of Candida to them, and the fact that PDT can treat recurring infections caused by drug-resistant Candida, this is one of the advantages of PDT over azoles.

For the treatment of candidiasis, the use of topical antifungal agents provides temporary relief, but recurrence is a common problem, especially in the case of immunodeficiency [21]. Mima [19] et al. showed recurrence of palatal inflammation in 75% and 78% of patients in the NYT and PDT groups, respectively, during follow-up, Scwingel [33] et al. showed recurrence of signs and symptoms in 72% of patients in the control group on day 30, and Macial [21] et al. found recurrence in 25% of patients in the experimental group and 12.5% in the control group, which may be related to improper denture cleaning and Candida recolonization in patients with denture stomatitis. With regard to safety, no adverse reactions were reported in Macial [21], while there were varying degrees of nausea and burning tongue in the studies of Yue Zhao [31] and Jinmei Tan [32], which may be related to immunodeficiency in HIV-infected patients.

Due to the widespread use of antifungal drugs such as nystatin and drug resistance in some patients, the clinical effectiveness of their treatment of oral candidiasis still needs to be improved [42]. PDT has a wide antibacterial spectrum, short therapeutic course and strong targeting, and can cause death of Candida by changing the permeability of Candida [43]. Combining the two methods may have more significant effects, so it is of great clinical significance to explore the combined application of PDT and mycobacterium. The results of this Meta-analysis showed that the combination of PDT and nystatin was more effective than nystatin alone in the treatment of oral candidiasis, and the recurrence rate was lower, which may be related to the mechanism of fungal inactivation by PDT interacting with the mechanism of antifungal drugs. Nystatin is a polyene antibiotic that interacts with ergosterol in fungal cell membranes, making them porous and susceptible to cracking, thus exerting its antifungal action [44]. PDT, on the other hand, is a photochemical reaction to excite a photosensitizer to produce reactive oxygen species, which can react with a variety of biomolecules such as proteins and phospholipids of fungal cells to produce activity and eventually inactivate the cells [45]. Therefore, the synergistic effect of the two treatment measures makes the combination more effective than a single antifungal drug. Regarding the safety of the combination therapy, the results of the Chen Chong [29] study showed that the incidence of adverse reactions during treatment was similar in both groups, and the adverse reactions resolved on their own without treatment, suggesting that the combination therapy was safe and reliable. The combination of PDT and antifungal drugs is recommended in clinical treatment and may be a more reliable measure for reducing the recurrence of oral candidiasis.

Smoking was identified as one of the important risk factors for increased oral Candida carriage in the included studies [46]. A meta-analysis by Nader [47] showed that smokers had significantly higher rates of oral Candida carriage than non-smokers. Smoking reduces the activity of oral leukocytes, decreases gingival exudate, and reduces the load of immunoglobulins and leukocytes, thus contributing to the colonization of Candida in the oral cavity [48]. Abduljabbar [38] conducted a clinical trial on the presence of smoking as a risk factor in patients with denture stomatitis and found that PDT was significantly more effective in non-smokers than in smokers.

This Meta has some limitations to consider, the lack of an appropriate number of RCTs included in each subgroup, as well as the small sample size included in some studies and the differences in follow-up time between studies to the extent that bias may result in subgroup analysis. There is a lack of standardization in the use of PDT across studies, such as differences in photosensitizers, activation wavelengths, power output, irradiation duration, and energy dose. In addition, the choice of the optimal synergistic treatment modality of PDT with other drugs still needs to be studied in depth.

## Conclusion

PDT was effective in the treatment of oral candidiasis; PDT was more effective than nystatin for the treatment of denture stomatitis in the palate, while there was no significant difference between the two for the denture site; The efficacy of PDT for oral candidiasis was similar to that of fluconazole; PDT was less effective than miconazole for oral candidiasis; Compared with nystatin alone, the combination of PDT and nystatin is more effective in treating oral candidiasis with less risk of recurrence.

### Supplementary Information

**Additional file 1. **

## Data Availability

All data generated or analyzed during this study are included in this article.

## Abbreviations

PDT: Photodynamic Therapy
OC: Oral Candidiasis
DS: Denture stomatitis
ROS: Reactive Oxygen Species
DNA: Deoxyribonucleic Acid
HIV: Human Immunodeficiency Virus
CFU: Colony forming units
RR: Relative Risk
MD: Mean Difference
CI: Confidence Interval
FEM: Fixed-effects Model
REM: Random-effects Model
PRISMA: Preferred Reporting Item for Systematic Reviews and Meta-analyses
RCT: Randomized Controlled Trial
Rb: Rose Bengal
Cur: Curcumin
PDZ: Photosensitive Diazine
MB: Methylene Blue
DMMB: Dimethyl Methylene Blue
ICG: Indocyanine Green
NYT: Nystatin
LPL: Low-power laser
LLLT: Low-level laser therapy
GRADE: Grading of Recommendations Assessment, Development and Evaluation
